# HIV risk perception and behavior among medically and traditionally circumcised males in South Africa

**DOI:** 10.1186/s12889-016-3024-y

**Published:** 2016-04-26

**Authors:** N. P. Zungu, L. C. Simbayi, M. Mabaso, M. Evans, K. Zuma, N. Ncitakalo, S. Sifunda

**Affiliations:** Human Sciences Research Council, Office of the CEO, Private Bag X41, Pretoria, 0001 South Africa; University of Cape Town, Cape Town, South Africa; Health Systems Trust, Durban, South Africa

**Keywords:** HIV, Risk perception, Risk behavior, Male circumcision

## Abstract

**Background:**

In South Africa, voluntary medical male circumcision (VMMC) has recently been implemented as a strategy for reducing the risk of heterosexual HIV acquisition among men. However, there is some concern that VMMC may lead to low risk perception and more risky sexual behavior. This study investigated HIV risk perception and risk behaviors among men who have undergone either VMMC or traditional male circumcision (TMC) compared to those that had not been circumcised.

**Methods:**

Data collected from the 2012 South African national population-based household survey for males aged 15 years and older were analyzed using bivariate and multivariate multinomial logistic regression, and relative risk ratios (RRRs) with 95 % confidence interval (CI) were used to assess factors associated with each type of circumcision relative no circumcision.

**Results:**

Of the 11,086 males that indicated that they were circumcised or not, 19.5 % (95 % CI: 17.9–21.4) were medically circumcised, 27.2 % (95 % CI: 24.7–29.8) were traditionally circumcised and 53.3 % (95 % CI: 50.9–55.6) were not circumcised. In the final multivariate models, relative to uncircumcised males, males who reported VMMC were significantly more likely to have had more than two sexual partners (RRR = 1.67, *p* = 0.009), and males who reported TMC were significantly less likely to be low risk alcohol users (RRR = 0.72, *p* < 0.001).

**Conclusion:**

There is a need to strengthen and improve the quality of the counselling component of VMMC with the focus on education about the real and present risk for HIV infection associated with multiple sexual partners and alcohol abuse following circumcision.

## Background

In South Africa, it is estimated that a widespread roll-out of voluntary medical male circumcision (VMMC) may prevent up to two million HIV infections and 300,000 deaths over a 10-year period [1]. In addition to VMMC, parts of South Africa have a long history of traditional male circumcision (TMC) [9, 10]. Among some ethnic groups TMC is seen as a way of entrenching social norms, engendering responsibility and morality, and imparting cultural knowledge [2]. Although no randomised control study has been done to demonstrate this, there is ample ecological evidence from several studies that also suggests that TMC reduces the risk for male HIV infection through heterosexual transmission [3, 4].

One of the major concerns about the promotion of male circumcision in general and VMMC in particular as an HIV prevention strategy has been the fact that this may lead to the perception of circumcision as a ‘natural condom’ [5–7]. There is therefore a possibility of low risk perception among circumcised leading them to engage in risky sexual behavior such as non-condom use with high-risk partners and multiple sexual partners, and this is commonly known as risk compensation [8–10]. Evidence from simulation studies suggest that risk compensation has a potential to reduce the impact of VMMC interventions on HIV incidence [11, 12]. Some studies have even suggested that a modest level of risk compensation in men could increase female HIV infections [13].

Studies on risk compensation following the randomized VMMC trials conducted in South Africa, Uganda and Kenya have provided a mixed picture. A South African study [14] found risk compensation among the intervention group of circumcised males who had an increased number of sexual acts. However, this increase in risky behavior was not found to reduce the protective effect of male circumcision against HIV infection. Studies from Uganda have shown risk reduction and protection up to 5 years following the circumcision trials [15], while evidence from Kenya found no association between risk compensation and male circumcision from one year after baseline [16].

In South Africa, studies on traditionally circumcising populations and risk compensation have also found conflicting results. One study found evidence of risk compensation among those who had heard about the protective benefits of VMMC [17], while another found men who had heard that circumcision reduces a man’s risk of HIV infection to have even greater risk perceptions and an increased likelihood of condom use [18]. Relatively little is known about the differences in risk compensation for those who are traditionally circumcised compared to those who are medically circumcised. Yet in some communities the two main types of male circumcision continue to be done side-by-side in many African countries including South Africa [19, 20].

Understanding the relationships between type of circumcision, risk perception and sexual risk behaviors is important for strengthening the protective effects of male circumcision. The aim of the paper was to explore HIV risk perceptions and risk behavior among those who have undergone either VMMC or TMC when compared to those who have not been circumcised.

## Methods

The South African 2012 national population-based household survey was conducted using second-generation surveillance survey approach. The survey methodology is described in detail elsewhere [21]. Briefly, a multistage stratified systematic probability sample of 15 visiting points (VPs) or households was drawn from each of the randomly selected 1000 enumeration areas (EAs) sampled from 86,000 EAs based on the 2001 census EAs. The selection of EAs was stratified by province and locality type defined as urban formal, urban informal, rural formal (including commercial farms), and rural informal localities. Out of 15,000 VPs that were sampled 13,083 were found to be valid. Persons of all ages living in selected South African households and hostels were all eligible to participate in the study. A total of 42,950 individuals in the valid households were eligible to be interviewed, and 38,431 agreed to be interviewed and 28,997 out of these agreed to provide blood specimens for HIV testing [21].

A detailed questionnaire soliciting information related to demographic characteristics, knowledge, attitudes, practice, behaviors and circumcision type was administered. This analysis is based only on adult data, and the analysis focused on males aged 15 years and older who participated in the survey. All youth and adults who participated provided either written or verbal consent, including parent/guardian informed consent for youth under 18 years of age and youth verbal assent to have a blood specimen taken

The survey protocol was approved by the Human Sciences Research Council’s Research Ethics Committee (REC: 5/17/11/10) as well as by the Associate Director of Science of the National Centre for HIV and AIDS, Viral Hepatitis, STD and TB Prevention at the USA’s Centers for Disease Control and Prevention (CDC) in Atlanta.

### Measures

The data was used to extract information on males who were circumcised (either medically or traditionally), and those who were not circumcised. Therefore the primary outcome variable has three levels, VMMC, TMC and no circumcision, which makes it a multinomial outcome.

Demographic measures controlled for in the analysis included age (15–19, 20–24, 25–49, 50 years and older, race (Black Africans or other races), locality type (urban formal, urban Informal, rural informal, rural formal areas) and province (Western Cape, Eastern Cape, Northern Cape, Free State, KwaZulu-Natal, North West, Gauteng, Mpumalanga, Limpopo). Explanatory variables included questions on self-perceived risk of HIV (no or yes), and sexual behaviors which included sexual debut (<15 or ≥15 years of age), number of sexual partners in the last 12 months (one partner, two partners or more than two partners), condom use at last sex with the most recent sexual partner (no or yes), and alcohol use based on Alcohol Use Disorder Identification Test (AUDIT) scores [22].

### Statistical analysis

Descriptive statistics were used to summarize demographic characteristics, HIV risk perception, and risky behavior by circumcision type, and differences between categorical variables were assessed using Chi-square test. Bivariate and multivariate multinomial logistic regression models were used to assess factors associated with each type of circumcision using no circumcision group as a reference category. The relative risk ratio (RRR) computed as the exponentiated coefficient from -*mlogit*- was used as a measure of association and reported with 95 % confidence intervals (CI) and a *p* ≤ 0.05. The “svy” command was used to take into account complex design of the survey. All statistical analysis was conducted using Stata software version 11 (Stata Corp., College Station, TX, USA).

## Results

### Demographic characteristics and circumcision status

Of the 11,086 males in the study, 19.5 % (95 % CI: 17.9–21.4) were medically circumcised, 27.2 % (95 % CI: 24.7–29.8) were traditionally circumcised and 53.3 % (95 % CI: 50.9–55.6) were not circumcised. Study participants were not obliged to respond to all questions and therefore total responses did not always add up to the total.

There was a statistically significant difference in status and type of circumcision by age, race, locality type and province (Table 1). VMMC was the most commonly reported type of circumcision among adolescents (15–19 years old) than TMC (20.1 % versus 13.3 %), and inversely TMC was most commonly reported among adults 25 years and older. Black Africans reported more TMC than other race groups (33.9 % versus 19.1 %), and the opposite was true for VMMC (20.9 % versus 3.9 %). Man from urban formal areas reported more VMMC while those rural informal and urban informal areas reported more TMC. Gauteng province (GP) had the highest percentage of males who were medically circumcised (29.8 %), while Northern Cape (NC) (10.6 %) had the lowest percentage of males who were medically circumcised. In contrast, Eastern Cape (EC) had the highest percentage of males who were traditionally circumcised (64.9 %) and KwaZulu-Natal (KZN) had the lowest percentage of males who were traditionally circumcised (6.9 %).

**Table 1:** Demographic characteristics by male circumcision type among males 15 years and older

Variables		Voluntary medical male circumcision (VMMC)	Traditional male circumcision (TMC)	No circumcision	
	n	% (95 % CI)	% (95 % CI)	% (95 % CI)	*p*-value
Age group (in years)					
15–19	1.778	20.1 (16.6–24.2)	13.3 (10.5–16.8)	66.6 (62.3–70.6)	<0.001
20–24	1.585	22.8 (19.25–26.8)	24.5 (20.6–28.8)	62.7 (48.4–57.0)	
25–49	4.785	20.1 (17.8–22.6)	30.2 (27.0–33.6)	49.7 (46.5–52.9)	
50+	2.938	15.0 (12.7–17.6)	31.9 (28.4–35.6)	53.2 (49.5–56.8)	
Race					
Black African	6.255	19.1 (17.0–21.4)	33.9 (30.9–37.1)	47 (44.1–49.9)	<0.001
Other	4.798	20.9 (18.5–23.6)	3.9 (2.90–5.3)	75.2 (72.2–77.9)	
Locality type					
Urban formal	6.528	24.1 (22.0–26.5)	18.4 (15.5–21.8)	57.4 (54.5–60.3)	
Urban informal	1.152	14.0 (10.6–18.3)	39.2 (32.6–46.2)	46.9 (40.2–53.7)	
Rural informal	2.164	14.7 (12.1–17.7)	39.5 (34.1–45.2)	45.8 (40.4–51.3)	
Rural formal	1.242	14.8 (6.8–29.3)	18.1 (11.5–27.2)	67.1 (58.8–74.5)	
Province					
Western Cape	1.356	17.7 (14.4–21.6)	22.3 (15.3–31.5)	60.0 (53.1–66.4)	<0.001
Eastern Cape	1.395	10.9 (7.1–16.4)	64.9 (57.9–71.4)	24.2 (20.0–29.0)	
Northern Cape	896	10.6 (7.8–14.2)	9.6 (5.5–16.2)	79.8 (73.4–84.9)	
Free State	848	17.1 (12.4–23.1)	19.7 (14.7–25.9)	63.2 (56.5–69.4)	
KwaZulu-Natal	2.532	16.2 (12.9–20.1)	6.9 (4.9–9.4)	77.0 (72.9–80.6)	
North West	780	16.7 (13.5–20.5)	19.5 (14.3–26.0)	63.8 (57.6–69.6)	
Gauteng	1.492	29.8 (26.1–33.9)	18.0 (14.3–22.3)	52.2 (48.3–56.1)	
Mpumalanga	858	11.4 (8.2–15.7)	39.8 (29.3–51.4)	48.8 (38.1–59.6)	
Limpopo	929	25.8 (21.4–30.6)	49.4 (42.7–56.0)	24.9 (19.4–31.3)	

Furthermore, there was a statistically significant difference between status and type of circumcision and perceived risk of HIV infection, number of sexual partners, and alcohol use but no difference was found with sexual debut and condom use at last sex (Table 2). More traditionally circumcised males perceived themselves not at risk of HIV infection compared to those who thought of themselves at risk of HIV (33 % versus 25 %), and the opposite was true for VMMC. Traditionally circumcised males reported more multiple sexual partners in the last 12 months compared to those circumcised through VMMC. Similarly, traditionally circumcised males reported more alcohol used than those circumcised through VMMC.

**Table 2:** HIV risk perception and sexual risk behavior by circumcision type among males 15 years and older

Variables		Voluntary medical male circumcision (VMMC)	Traditional male circumcision (TMC)	No circumcision	
	n	% (95 % CI)	% (95 % CI)	% (95 % CI)	*p*-value
Perceived risk of HIV infection					
No	1.853	15.8 (13.1–19.1)	33.2 (29.1–37.6)	51.0 (46.5–55.4)	<0.001
Yes	9.143	20.5 (18.7–22.4)	25.5 (22.9–28.3)	54.0 (51.6–56.4)	
Sexual debut (age in years)					
<15	275	18.2 (12.1–26.5)	26.8 (18.5–37.2)	54.9 (45.1–64.4)	0.080
≥15	3.068	21.9 (18.8–25.3)	17.8 (15.2–20.8)	60.3 (56.8–63.7)	
Condom use at last sex					
No	4.648	20.3 (17.8–23.0)	28.5 (25.5–31.8)	51.2 (48.0–54.3)	0.471
Yes	2.352	21.1 (18.2–24.3)	30.4 (26.7–34.4)	48.5 (44.7–52.3)	
No. of sex partners in last 12 months					
One partner	6.020	19.6 (17.5–21.9)	29 (26.1–32.1)	51.4 (48.5–54.2)	0.050
Two partners	614	22.7 (17.5–28.8)	29.5 (23.8–35.9)	47.8 (41.6–54.2)	
More than 2 partners	514	25.6 (20.3–31.6)	33.5 (26.6–41.1)	41.0 (34.4–47.9)	
Alcohol use risk score^a^					
Abstainers	4.472	17.8 (15.8–20.1)	29.1 (26.1–32.3)	53.1 (49.8–56.4)	0.026
Low risk (1–7)	3.421	22.3 (19.5–25.3)	25.3 (21.9–28.9)	52.5 (49.0–56.0)	
Risky/hazardous level (8–15)	1.371	22.3 (18.3–27.0)	22.9 (19.1–27.2)	54.8 (50.3–59.2)	
High risk/harmful (16–19)	254	20.3 (13.2–29.9)	34.6 (25.8–44.7)	45.1 (36.1–54.3)	
High risk (20+)	234	23.9 (15.8–34.4)	25.9 (18.5–35.0)	50.2 (40.1–60.3)	

^a^Risk score based on a questionnaire for Alcohol Use Disorder Identification Test (AUDIT)

### Multivariate model

The final multivariate model in Table 3 shows independent associations between a combination of factors with VMMC and TMC relative to those who reported no circumcision. The direction of the associations for all variables is similar to the directions that were observed in the univariate analyses. However, the effects or the magnitude of associations vary slightly but in the same direction.

**Table 3:** Multinomial multivariate logistic regression analyses of socio-demographic factors, HIV risk perception and risky behavior associated with male circumcision type compared to non-circumcised males

Variables	Voluntary medical male circumcision (VMMC)			Traditional male circumcision (TMC)		
	RRR	95 % CI	*p*-value	RRR	95 % CI	*p*-value
Age group (in years)						
15–19	1			1		
20–24	1.20	0.76–1.90	0.421	2.24	1.54–3.27	<0.001
25–49	1.51	0.95–2.40	0.085	3.54	2.50–5.00	<0.001
50+	1.34	0.82–2.20	0.243	5.15	3.56–7.46	<0.001
Race						
Other	1			1		
Black Africans	2.23	1.41–3.52	0.001	25.44	19.41–33.35	<0.001
Locality type						
Urban formal	1			1		
Urban informal	0.74	0.48–1.14	0.169	1.68	1.34–2.11	<0.001
Rural informal	0.85	0.60–1.21	0.377	1.27	1.01–1.59	0.042
Rural formal	0.77	0.32–1.82	0.551	1.10	0.85–1.42	0.458
Province						
Western Cape	1			1		
Eastern Cape	2.03	1.01–4.10	0.048	2.57	1.87–3.54	<0.001
Northern Cape	0.38	0.22–0.65	<0.001	0.10	0.07–0.16	<0.001
Free State	0.73	0.37–1.43	0.359	0.18	0.13–0.25	<0.001
KwaZulu-Natal	0.41	0.26–0.67	<0.001	0.05	0.03–0.07	<0.001
North West	0.58	0.33–1.01	0.054	0.14	0.10–0.20	<0.001
Gauteng	1.22	0.83–1.77	0.308	0.26	0.19–0.36	<0.001
Mpumalanga	0.62	0.34–1.12	0.111	0.28	0.20–0.39	<0.001
Limpopo	2.85	1.49–5.42	0.001	0.71	0.50–1.02	0.061
Perceived risk of HIV infection						
No	1			1		
Yes	1.35	0.99–1.85	0.059	1.02	0.85–1.22	0.861
No of sex partners in last 12 months						
One partner	1			1		
Two partners	1.02	0.71–1.48	0.896	0.79	0.61–1.03	0.088
More than 2 partners	1.67	1.14–2.46	0.009	1.21	0.91–1.62	0.188
Alcohol use risk score^a^						
Abstainers	1			1		
Low risk (1–7)	1.28	0.94–1.76	0.121	0.72	0.60–0.86	<0.001
Risky/hazardous level (8–15)	1.02	0.74–1.41	0.891	0.81	0.65–1.01	0.064
High risk/harmful (16–19)	1.29	0.68–2.42	0.434	0.94	0.62–1.43	0.768
High risk (20+)	1.19	0.62–2.29	0.606	0.70	0.43–1.12	0.132

*RRR* relative risk ratio equivalent to odds ratio, *CI* confidence intervals

^a^Risk score based on a questionnaire for Alcohol Use Disorder Identification Test (AUDIT)

Relative to males who reported that they were not circumcised older generation (males older than 20 years) were significantly more likely (*p* < 0.001) to report TMC than adolescents 15–19 years old. Black African males were significantly more likely to report both VMMC (RRR = 2.23, *p* = 0.001) and TMC (RRR = 25.44, *p* < 0.001) than other race groups. The likelihood of reporting TMC was significantly higher among males living in urban informal (RRRs = 1.68, *p* < 0.001) and rural informal settings (RRR = 1.27, and *p* < 0.042).

Relative to the WC Province, males in the EC were significantly more likely to report both VMMC (RRR = 2.03, *p* = 0.048) and TMC (RRRs =2.57, *p* < 0.001). The opposite was true for NC (RRR = 0.38, *p* < 0.001 and RRR = 0.10, *p* < 0.001), KZN (RRR = 0.41, *p* < 0.001 and RRR = 0.05, *p* < 0.001) and NW (RRR = 0.58, *p* = 0.54 and RRR = 0.14, *p* < 0.001) with regards to reporting of VMMC and TMC, respectively. Reporting of TMC was significantly less likely among males in FS (RRR = 0.18, *p* < 0.001), GP (RRR = 0.26, *p* < 0.001) and MP (RRR = 0.28, *p* < 0.001), while males in LMP were significantly more likely to report VMMC (2.85, *p* = 0.001).

No statistically significant associations were found between circumcision type (VMMC and TMC) the perceived risk of HIV, sexual debut, and condom use at last sex. The final model showed that relative to uncircumcised males, males who reported VMMC were significantly more likely to have had more than two sexual partners (RRR = 1.67, *p* = 0.009). Furthermore, males who reported TMC were significantly less likely to be low risk alcohol users relative to uncircumcised males (RRR = 0.72, *p* < 0.001).

## Discussion

The study found that after controlling for age, race, locality type and province HIV risk perception was not independently associated with either type of circumcision. There was also no evidence that undergoing VMMC or TMC influenced the risk behavior of males with regards to the age of sexual debut when comparing them with males who were not circumcised. Additionally there were no differences between the two groups with regards to condom use at last sexual encounter when compared with males who had not been circumcised.

The findings on risk compensation in this study are similar to previous studies that also did not find any strong evidence of risk compensation among men who had undergone VMMC. In a study conducted in Uganda one Randomized Controlled Trial (RCT) found very little evidence of behavioral disinhibition or risk compensation among men who had undergone circumcision [23]. Mattson et al. [16] utilized a different measure of risky behavior a risk scale, and found no significant differences in their 18-item sexual risk propensity scores between circumcised and uncircumcised men.

There are many factors that may offset risk compensation among men who have undergone male circumcision and that could explain why this study did not find any significant associations between circumcision type and risk perception. Men who decided to undergo VMMC for HIV prevention are exposed to accompanying interventions of pre- and post-HIV test counseling as well as circumcision-related pre- and post-test counseling procedure [24]. This provides an opportunity for health education and promotion to reduce HIV transmission, and to deter assumptions about circumcision as an ‘invisible condom’. Males who seek VMMC for HIV prevention may also do so because they perceive themselves to be at risk of HIV infection, whereas males undergo TMC for cultural reasons [25, 26]. Indeed, the stagnant rates of TMC in South Africa [21] compared to increasing rates of VMMC suggests that Black African males are likely to undergo TMC for cultural reasons and not because they think circumcision will reduce their risk of contracting HIV. This may explain the lack of independent associations between TMC, risk perception and risk behavior.

This study points to possible shortcomings in the HIV risk reduction counselling interventions that are provided as part of VMMC in the clinical setting. Desired outcomes of VMMC counselling include behavior change to increase protective behavior, and reduce risk of HIV among men who undergo VMMC [25]. Current findings show evidence of a relationship between VMMC and multiple sexual partners, where according to the multivariate model the risk of having more than two sexual partners was higher among males who had undergone VMMC compared to uncircumcised men, but not for males who had TMC. This is of great concern since it is expected that this group would have been exposed to some risk reduction education and counseling which is aimed at reducing risky sexual behavior as part of the VMMC procedure. Males who reported TMC were less likely to be alcohol users, and although not statistically significant males who reported VMMC were more likely to be risky alcohol users. This warrants a reexamination of information and/or messages imparted among during VMMC procedure with a focus on multiple sexual partnership and alcohol abuse.

While the use of a nationally representative sample and the large sample size are clear strengths of this study which allow for meaningful analyses of the data that enables results to be generalised to the whole South African population, as with all research studies there are also some limitations. One of the major limitations of the study is that the measures of both HIV risk perception and HIV risk behaviors are based on participants self-report and these may be affected by recall and social desirability bias. The cross sectional nature of the study is limited to assessing associations and cannot infer causality.

## Conclusion

The current findings do not suggest that there is HIV risk compensation among men who undergo VMMC with respect to risk perception, sexual debut, and condom use at last sex. However, the finding of higher risk of multiple sexual partners and indication of alcohol abuse among males who had VMMC is worrisome. This highlights the need to strengthen and improve the quality of the counseling component of VMMC with the focus on education about the real and present risk for HIV infection associated with multiple sexual partners and alcohol abuse following circumcision.

### Availability of data and materials

The dataset(s) supporting the conclusions of this article is currently not available in the public domain. The data will be curated and accessed through the Human Sciences Research Council data research repository via access dataset http://www.hsrc.ac.za/en/research-data/ by the end of 2016.
